# Contagion and Interpersonal Influence: Distinguishing Mechanisms of Behavior Change Using Social Network Theory

**DOI:** 10.21307/connections-2019.041

**Published:** 2024-08-20

**Authors:** Thomas W. Valente

**Affiliations:** Department of Population & Public Health Sciences, Keck School of Medicine, University of Southern California

**Keywords:** Network Effects, Diffusion of Innovations, Social Influence, Contagion

## Abstract

The present paper articulates the many ways social network researchers conceptualize and operationalize network influences on people’s behaviors. Four categories of network influences are described: (1) personal network, (2) positional, (3) network-level, and (4) individual network-level interactions. Personal network effects are based on data from the individual’s direct and indirect contacts. Positional effects are derived from the individual’s position in the network such as being central or peripheral. Network-level effects are measured using network-level indicators such as centralization or clustering. Interaction effects occur when there is consideration of both the individual and network level measures such as understanding the influence of being in a central position in a centralized or decentralized network. These various network effects are contrasted with contagion, which is the most frequently used mechanism for diffusion. One conclusion drawn from this review is that when we invoke contagion explanations, we give agency to the product, whereas when we invoke interpersonal influence explanations, we give agency to people and social systems.

## Introduction

Considerable research has been conducted to show that certain behaviors are contagious and spread through person-to-person contact. Often this research is informed by diffusion of innovations theory (Rogers, 2003) and, in other cases, as social movements (Diani & McAdam, 2003), marketing (Van den Bulte & Wuyts, 2007), or persuasion theory (Cialdini & Goldstein, 2002). Contagion, as defined by Merriam-Webster, is the transmission of a disease by direct or indirect contact and also an influence that spreads rapidly. Contagion is used both in the context of disease spread and interpersonal influence usually implying direct contact. Interpersonal influence, however, can occur through a wide variety of mechanisms that social network theory has articulated in empirical studies over the past 60 or so years. The purpose of this article is to review the many social influence mechanisms that have been developed from the social network perspective to expand our understanding of influence beyond direct contact. Influence in this context refers to when an individual makes attitudinal, behavioral, or other changes in response to others in the social network.

There are many ways that interpersonal influence can be transmitted via social networks. Behavior characteristics, cultural norms, and/or settings may help determine which of these different mechanisms occurs. One of the fundamental challenges for network researchers is to test the appropriate mechanism so that the existence of network effects can be estimated confidently. We do not provide an introduction or overview of social network theory or methods, as there are several alternative and comprehensive sources for this (Borgatti et al., 2013; Kadushin, 2012; Luke & Harris, 2007; Smith & Christakis, 2008; Valente, 2010; 2015). In addition, it is acknowledged that many studies have been conducted to test for contagion (Aral et al., 2009; Fowler et al., 2011; Shalizi & Thomas, 2011) and find it challenging to demonstrate it definitively. This paper further muddies the picture by articulating the array of different influence mechanisms beyond mere contagion. Nonetheless, we offer a path through the mud that is productive for those attempting to apply network theory to understand behavior change.

**Figure 1:**
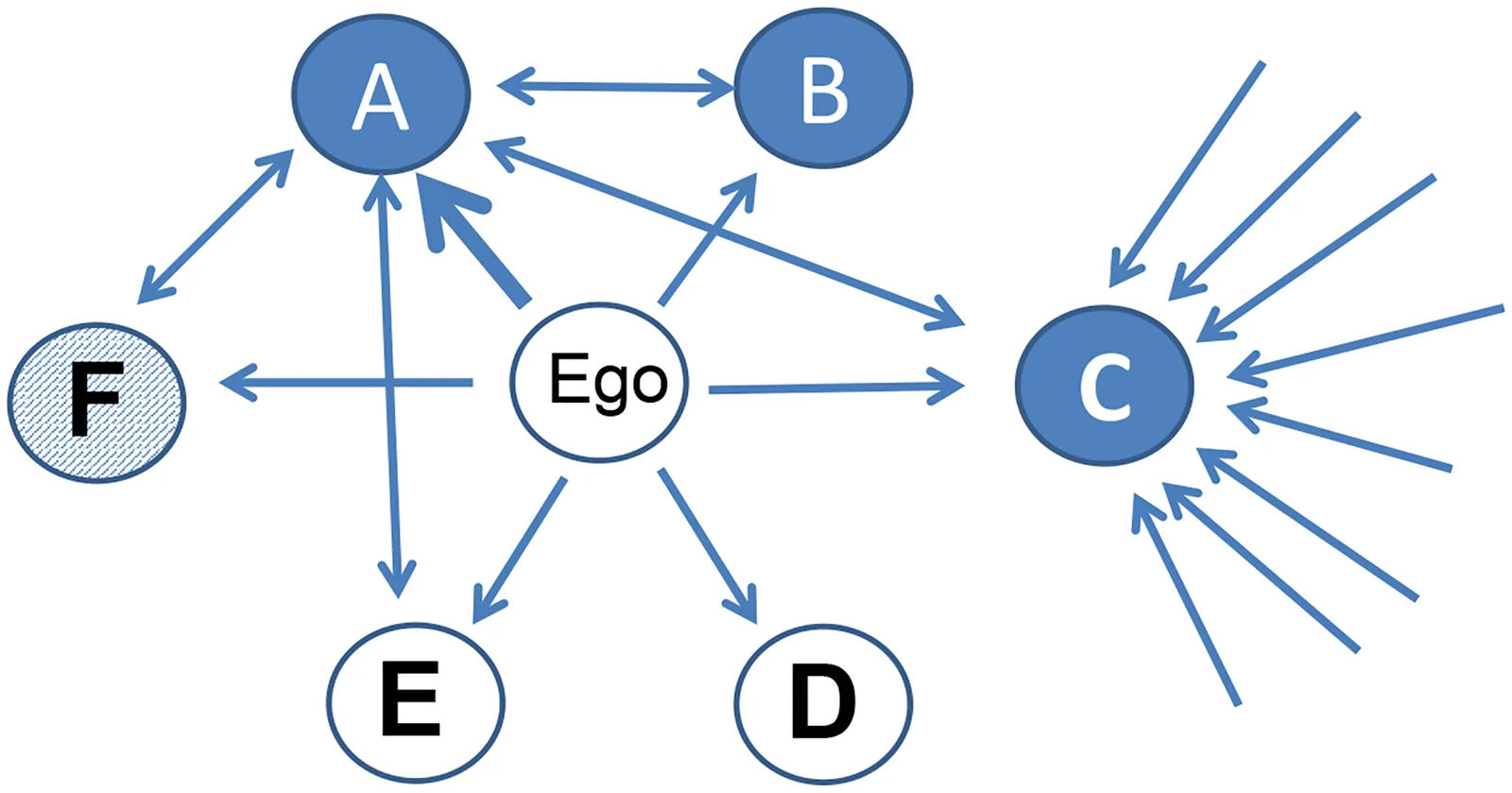
Personal network of ego who is connected to six alters, three of whom are adopters (A, B, & C); and F is perceived to use; D and E are nonadopters. Arrows reflect tie direction with the tie between ego and A being stronger than the others. C receives many ties from outside of the ego’s personal network

The first section of the paper will address personal network effects, that is, those that occur within or through an individual’s immediate or personal network as illustrated in Figure 1. We further delineate this section into personal network exposure, exposure weighted by tie characteristics, and exposure weighted by alter characteristics. The second section addresses positional effects, that is, how an individual’s position within a network may influence behavior. The third section three discusses network-level effects, and the final section addresses the interaction between the personal and position effects within the context of network-level influences. For example, if a central person in a centralized network behaved differently than a central person in a decentralized network, this would be a position network-level interaction. In this article, the emphasis is on network structural mechanisms of influence rather than the type of communication or influence that is transmitted by the network. In other words, when describing a network structural mechanism, there is a de-emphasis on the type of communication or influence that occurs through such means as normative pressure, peer modeling, mimicry, information sharing, or some other mechanism. Instead, we emphasize the network structural elements of influence.

## Ego-Centric or Personal Network

### Exposure

#### Personal Network Exposure

The most obvious and basic personal network influence is exposure. Individuals with personal networks composed of a majority of adopters are more likely to subsequently engage in the behavior of study. Adopters are people who use new products or start new behaviors such as becoming an e-cigarette user, downloading a new app, or purchasing certain consumer products. In Figure 1, ego is connected to six alters four of whom are adopters. Thus, ego would be expected to become an adopter (assuming roughly equal influence from all alters). Exposure is clearly the structural mechanism most closely associated with the concept of contagion. One significant difference, however, is that contagion generally assumes that mere contact is sufficient for transmission, whereas exposure often implies that a majority of adopters increases the likelihood of adoption. In addition, it is often assumed that there is a dose–response relationship between exposure and adoption such that each additional alter adopter increases the likelihood of adoption.

Exposure is most frequently measured via perceived peer approval or use. Hundreds of studies include peer exposure as a proxy for perceived norms. Although an intuitive concept, the first documented analysis of personal network exposure would be Rogers’ (1979) chapter analyzing the association of networks with family planning use among rural Korean women in 25 villages during the 1960s and early 1970s (Rogers & Kincaid, 1981). Coleman et al. (1966) estimated contagion using a network positional explanation for physician adoption of a new drug, not by calculating exposure. They showed that diffusion was more rapid among physicians with more contacts compared to those with fewer (but see Van den Bulte & Lillien, 2001 for a marketing explanation).

#### Perceived Use

Perceived use, when ego thinks his/her alters are adopters regardless of those alter self-reports, has been shown to be a stronger predictor than alter self-report use (Ianotti & Bush, 1992; Rice et al., 2003; Valente et al., 1997, 2012). There are many reasons why perceived use and alter self-report use may disagree: (1) alters tell ego they use when they do not; (2) ego assumes alters use without verifying they do; and (3) ego thinks alters do not use when they do. Regardless of why, it seems that humans are more strongly influenced by what they think is happening around them more so than what may actually be happening. This can occur because of projection or perceived norms; or can be a function of alter’s mis-representing their behavior or ego being uninformed of alter’s actual behavior.

#### Thresholds

If everyone waited until a majority of his/her network used before being willing to do so, nothing would diffuse (Valente, 1996). Some people must be willing to adopt before some or a majority of their peers do. Personal network thresholds are the proportion or number of others in one’s network a person requires to adopt a behavior before he/she is willing to adopt it. Thresholds, thus, are the variation in network exposure effects and range from zero to 100%, with low thresholds indicating people willing to adopt before their peers do and high thresholds signifying resistance to change or a desire to wait until most of their peers engage in the behavior before they do so.

Most low-threshold adopters are considered early adopters; however, some low-threshold adopters can be late adopters because their position in the network is such that they are exposed to the behavior late. Conversely, there can be some high-threshold adopters who are early adopters but late in their personal network. Thresholds may be similar for similar classes of products or ideas but could vary considerably between different types of products. The distribution of thresholds is thought to affect diffusion outcomes and rates (Valente & Vega-Yon, 2020).

#### Indirect Exposure

There is debate in the field as to whether interpersonal influence can be indirect. In their landmark paper on the spread of obesity within networks, Christakis and Fowler (2007) find evidence for indirect influence extending three steps from ego, the friend of a friend’s friend, for example. Valente (1995) posited that interpersonal influence may extend out two steps based on Friedkin’s (1998) observation that people are aware of the behaviors of their alters’ alters but no further, this so-called “horizon of observability.” Thus, it is not clear whether and to what extent indirect influences may occur in networks, and it is also unclear whether the intermediary nodes need to support the new practice in order for indirect influence to occur.

In addition, it may be possible to assign weights to these indirect influences. For example, Valente (1995) argued that indirect influence is inversely proportional to distance such that alters two steps from ego would have their influence divided by two, and those three steps away by three, and so on. It may also be the case that these potential indirect influences need to be weighted by the behavior of the intermediary alters, or by edge weights connecting the two step nodes to the one step ones and these to ego. Under this logic, it is possible that a strongly connected alter with a strongly connected alter could exert more influence on ego than a directly connected weak tie.

#### Structural Equivalence (SE)

**Figure 2:**
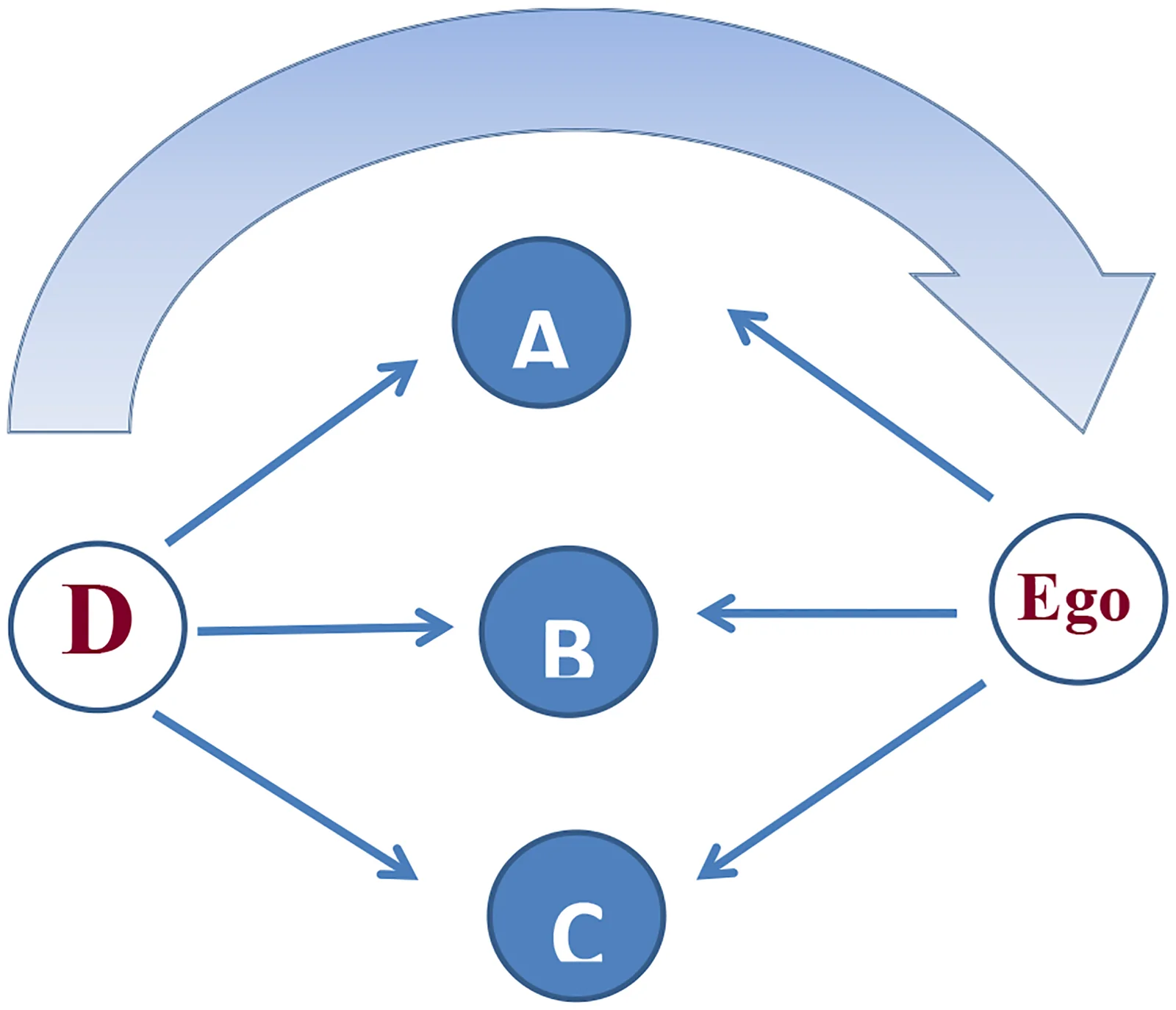
SE influence occurs when individuals, or other units, monitor the behavior of others who occupy the same or similar position in the network. Person D can influence Ego regardless of whether they are directly connected to one another. SE, structural equivalence.

Since at least 1979 (Sailer 1979), researchers have sought to devise measures that assess the extent to which two nodes in a network occupy similar positions. At the most basic level, two nodes are equivalent when they are connected to the same others, regardless of whether they are connected to each other. SE is the degree to which two nodes are equivalent from a network perspective and may also be conceived of as interchangeable. As Figure 2 illustrates, person D may influence ego because D and ego are connected to the same others and so potentially receive the same signals or advice from their network ties. There are several mechanisms by which SE may have its effects: (1) two SE nodes receive the same information because of their equivalent location in the network; (2) two SE nodes are in competition with one another and so monitor each other’s behavior (two competing firms for example); or (3) SE nodes are competing with one another for the attention of other network members (school kids vying for the attention of popular peers).

Burt (1987) published an influential paper arguing that the SE drives adoption decisions more so than cohesion (contagion or exposure via direct personal contacts). He illustrated this argument by re-examining the Medical Innovation data that Coleman et al. (1966) had collected among physicians in 1955–1957. One insightful adjustment Burt introduced in this analysis was to vary the weights attached to the influence of structurally equivalent alters. In other words, just as cohesion influences can be extended to indirect ties, ties of ties’ ties, and so on, SE influence could be weighted by giving more weight to alters based on their degree of SE. Thus, the diameter of influence can be contracted or expanded to match the researcher’s expectation about the strength of interpersonal influence whether based on cohesion or SE. From this view then, the researcher has two dimensions of interpersonal influence to consider: (1) cohesion vs. SE and (2) the social radius of that influence, whether narrow or broad.

It should also be noted that any network analytic technique that returns pairwise measures can be used to calculate exposures. For example, an n-clique analysis will return a matrix in which each cell indicates the frequency at which individuals share clique membership. Network exposures could then be calculated using this matrix and as such would model the rate individuals are exposed to an attitude or behavior by other network members with whom they share joint n-clique affiliations.

### Exposure Weighted by Tie Characteristics

#### Tie Strength

Granovetter’s classic strength of weak ties theory (Granovetter, 1973) emphasized how weak ties are strong in information because interpersonal communications between people who do not communicate frequently can be rich in the information they share. Weak ties, however, are strong in information, but not necessarily in influence. Because weak ties are infrequent communication partners, there is little opportunity for them to reinforce messages or reiterate persuasion attempts. Should ego adopt the behavior, the weak tie alters will not be readily available when ego has questions about how to implement the new practice or fix the technology when it breaks.

Strong ties, in contrast, are frequent communication partners, so communications about a new practice can be repeated and reinforced as the adoption process unfolds (Krackhardt, 1992; Valente & Vlahov, 2001). Indeed, it is quite likely that the more complex the behavior, the more likely the tie strength will be a factor in interpersonal influence. If the new practice is simply informational, such as a news item or rumor, ego can learn about it via weak ties. But if the new practice is complex and is a discontinuous innovation (e.g., the micro-computer, cell phone, solar panels), then ego is likely to be persuaded only by peers he/she trusts, sees frequently, and knows will be available when he/she has challenges with the innovation. Thus, for complex innovations, ego is more likely to be influenced by strong ties. In Figure 1, this indicates that ego is more likely to be influenced by alter A than B, as the thicker arrow from ego to A signifies a stronger tie than the other ones.

#### Reciprocity

Tie strength at times has been measured as reciprocity. That is, reciprocated ties are considered strong ones whereas un-reciprocated ties are considered weak. There are several limitations to this approach, however. First, influence stems from an individual’s perception of their social environment and individuals will model the behavior of those they nominate regardless of whether those nominations are reciprocated. Second, some relationships are inherently asymmetric, yet can still be influential. For example, who reports to whom in an organization is asymmetric, and in many occupational settings, one responds to their supervisor’s requests or suggestions (although clearly sometimes the influence can go in the other direction from lower to higher). Third, there are many instances in which data-collection methods may truncate the true network and hence record a reciprocated relationship as an un-reciprocated one. Reciprocated alter ties may exert stronger influence on ego for reasons other than tie strength. For example, individuals might decide to join a club only when reciprocated ties do so because they do not want to participate alone or in a situation in which they know no one.

#### Simmelian Ties

The sociologist Georg Simmel was one of the earliest scholars to note that interactions between two people can be strongly affected by the pair’s relations with other third parties. These so-called Simmelian ties (Krackhardt, 1999) affect ego’s reactions to alters and may affect the manner in which alters interact with ego. The presence of Simmelian ties among alters affects the influence alters have on ego. In general, alter influences are stronger when they have ties between them because their norms and behaviors reinforce one another. In Figure 1, A and B exert stronger influences on ego than C or F.

#### Personal Network Density

In addition to Simmelian tie influences among the triad of ego, A, and B, the configuration of all ties among ego’s neighbors can also affect their influence on ego. A person whose contacts are all connected to one another inhabits a personal world in which all communications are echoed and filtered by his/her network. The personal network is a tightly bound clique in which communications, norms, and persuasions are reinforced by everyone in his/her personal environment. Thus, once the idea becomes normative, ego has very little choice but to join the group’s behavioral decisions. In contrast, a person whose personal network has no Simmelian ties has a personal network in which the dyadic communications are not necessarily reinforced, and thus ego’s network has access to more diverse information (Burt, 2005). Indeed, some people may be comfortable having most or all their friends connected to one another, whereas others may prefer a set of distinct dyadic relationships so that interpersonal communications are strictly between ego and each alter. Empirical studies have confirmed that people are more strongly influenced by their network when it is dense rather than sparse (Centola, 2010; Kohler, 1997).

Simmelian ties and density influences are related, but distinct. Simmelian ties weight the influence of each alter based on how many ties the ego and alter share with the expectation that Simmelian ties exert stronger influence than non-Simmelian ones. Personal network density provides one measure based on the extent all alters are interconnected with the expectation that dense personal networks exert stronger influence than sparse ones. The source of influence is measured at different levels of analysis with each alter separately being weighted separately in Simmelian ties vs. all alters in aggregate with density.

### Exposure Weighted by Alter Characteristics

#### Alter Attributes

Researchers can consider the characteristics of the alters as well as the ego-alter dyad. Network influence may depend quite significantly on individual characteristics in at least four ways: egos are more likely to be influenced by (1) homophilous ties, alters that have the same characteristics as ego; (2) alters of higher status than ego; (3) alters having some specific characteristic such as being a boss, a team captain, or a coach, and (4) alters who have qualities that ego aspires to have. Depending on the setting and theoretical guidance, researchers may consider many different individual characteristics that might be associated with interpersonal influence. For example, for children homophily on sex may be an important consideration, whereas for adults, it might be homophily on socio-economic status.

#### Joint Participation

Interpersonal influences may also be a function of joint participation in activities or events. Network researchers have long-employed techniques using bipartite or two-mode graphs to measure joint participation in affiliations (Borgatti & Everett, 1997; Breiger, 1974). When data are available indicating which people belong to which groups, a network can be constructed measuring the frequency all pairs of individuals belonging to the same groups, or attending the same events. For example, monthly attendance at seminars can be recorded and a network constructed, which indicates the frequency at which all pairs of participants attended the same seminars. It may be that individuals are influenced by this joint participation perhaps because the pairs are exposed to the same information or because they are at the same place at the same time, they spend more time interacting with one another. For example, it has been shown that adolescents are more likely to be influenced by friends who are teammates than friends who are not (Fujimoto & Valente, 2013).

The calculation of networks via joint participation in events or activities provides a means to measure the influence of potential friends, or those with whom you have much in common but do not list as a friend. For example, among adolescents in schools, students may have others with whom they share many classes, clubs, and teams. These students will see each other often and are potentially influenced by them, yet they might not name each other as a friend, particularly if there is a limit on the number of friends one can name (Frank et al., 2008).

#### Network Indicator Weighted Influences

Although rarely used in empirical studies to date (Frank et al., 2004; Valente, 1995; Valente et al., 2015), network-weighted influences offer the potential to build network influence models matching the theory. For example, it may be that adolescents are influenced more by their peers who are popular (have many friends) than those who are not popular. If so, then the weight attached to the influence of alters should be proportional to the number of friendship nominations each person received. This would be a degree-weighted exposure model. This weighting can be incorporated into the influences discussed thus far such as SE, tie strength, indirect ties, and so on. In Figure 1, alter C receives many ties from others in the network and therefore may exert stronger influence on ego than the other alters.

There are dozens of specific centrality measures in the literature such as closeness, betweenness, eigenvector, and power that can be used to model specific influence processes based on the kinds of central people that could be expected to be more influential. Other individual measures may also be relevant though, such as weighting ties by individual measures of bridging (Valente & Fujimoto, 2010) or brokerage (Gould & Fernandez, 1989). More practically, one may want to weight alter influence based on whether ego and alter are members of the same network-defined group or cluster. For example, there are many ways to identify groups in networks (Girvan & Newman, 2002), and alter influences can be limited to dyads in the same group.

### Mediated

Throughout the discussion thus far, network ties have been described as face-to-face or in-person within a setting such as an organization, school, or community. Considerable interpersonal communication now occurs via media, generally referred to as social media, social networking, or online communication. This communication takes many forms, which we do not attempt to delineate here. The relevant question here is: Can interpersonal influence occur when mediated via communication technology? The answer seems to be a clear yes, with caveats, however. First, to date, the evidence seems to indicate that mediated interpersonal influences are weak (Bond et al., 2012; Centola, 2010; Ferrara & Yang, 2015; Huang et al., 2014). Second, although the influence may be weak, people can communicate with many others online and manage many more contemporaneous relationships online than is typically available face-to-face. On the other hand, the volume of contacts may lessen the influence of any one contact (Latané, 1981). Third, the opportunities for broadcasting or narrowcasting on these media open many possibilities for complex interpersonal influences to take place (Weng et al., 2012).

It should also be noted that this discussion of personal network exposure influences pertains primarily to sociometric network data in which all or many individuals are part of a bounded community such as an organization, school, community, online network, etc. Most network exposure measures can be calculated with ego-centric data provided there are alter–alter tie estimates except indirect exposures, SE, and the alter characteristic using a network measure.

In sum, there are a wide variety of interpersonal influence mechanisms that can be created using network theory, concepts, and tools. To be sure, having a theoretical justification for why a particular influence is being constructed is paramount; otherwise, researchers are embarking on a fishing expedition with no guidance as to what equipment to bring and where to find the fish. The vast number of influence weightings and options to expand or contract the radius of influence, and the real possibility of threshold effects, means that researchers can rarely be sure they have exhaustively tested all possible forms of interpersonal influence. In addition, a good practice is to conduct robustness checks such as when Burt (1987) varied SE exposures by varying the radius of observability. For example, robustness checks can be applied by repeating the analyses using alternative (theoretically justified) exposure measures.

Table 1 reports regression results designed to illustrate how these different network exposure weightings compare in one study. The data come from a longitudinal study of networks and smoking in five southern California high schools (Valente et al., 2013). Here, we regress smoking behavior on the lagged exposure values from the prior survey measurement including covariates for being female, academic grades, number of rooms in household, parental smoking, sibling smoking, being Hispanic/Latino, and fixed effects for school. Nine different network exposure weightings are included in separate regression equations.

The results show that most of the exposure variables are associated with smoking with the magnitude of the associations being similar. It is notable that the count variable is not associated with smoking, and SE has a negative association. While exposures are the most frequently employed network effects, it is also possible that actors engage in behaviors due to the position they occupy in the network.

## Network Position

Regardless of the behavior of one’s network peers, an individual’s position in the network may affect his/her behavior or time of adoption. We noted above that some people may be later adopters because their position in the network is such that they receive interpersonal exposure to the innovation late. Moreover, network position may also affect individuals’ perceptions of their role in the larger network. For example, peripheral members may feel unencumbered to conform to community norms and consequently innovate early or earlier than expected (Menzel, 1960).

### Centrality

The most obvious positional influence is being central in the network. Centrality may affect adoption timing in at least three ways. Central nodes: (1) have access to more information because they are in a privileged position, (2) can be constrained in their actions because they are aware many others may be monitoring their behaviors to determine what is normative, and (3) may feel pressure to act in order to retain their position of centrality. For example, popular adolescents in high smoking-prevalent schools may feel the pressure to start smoking early in anticipation that smoking will become normative in their school (Alexander et al., 2001).

There are at least a dozen centrality measures, many of which have various algorithms created for their implementation and calculation (Schoch ND). Specific centrality measures may suggest different influence mechanisms. For example, centrality in-degree is a count of the number of nominations received, whereas betweenness indicates the frequency at which a node lies on the shortest path connecting other nodes (Freeman, 1979). Degree centrality would most likely be associated with feeling the pressure to conform or engaging in behaviors in order to retain that central position. In contrast, betweenness centrality may affect adoption timing due to earlier access to information. Individuals with high power centrality may adopt behaviors in order to exert influence among others in the network.

In some empirical studies, popularity is measured by the number of times a person is nominated as a friend among in-school adolescents. This degree centrality has been associated with tobacco use. Degree centrality was positively associated with smoking in one study, and the interaction between degree and smoking prevalence was also associated with smoking (Alexander et al., 2001). In another study, in-degree and out-degree were separated, and empirical results showed that in-degree, frequency named as a friend, was associated with becoming a smoker whereas out-degree (number of friends named) was not (Valente et al., 2005). In the classic Korean family planning study, the contraceptive method of choice of most central women became the method of choice for a majority of the other women in their village (Rogers & Kincaid, 1981; also see Entwisle et al., 1996). Thus, if central nodes adopt a practice early, it can spread to other members of the network quickly (Valente & Davis, 1999).

### Peripheral or Marginal

Another potentially important position is being in a marginal or peripheral position. For example, data show that students on the margins of school-based friendship networks can be at higher risk for suicide ideation and suicide attempts. Sometimes, being on the margins of the network results in late access to information about new ideas and practices, thus resulting in late adoption. While being marginal may be associated with greater innovativeness (Menzel, 1960), it may also be disadvantageous when it comes to learning about what is happening within the community.

### Bridging

Network researchers have identified bridging actors in networks that act as nodes that connect otherwise-disconnected groups (Burt, 1992; Everett & Valente, 2016; Granovetter, 1973; Valente & Fujimoto, 2010). Bridging can provide access to the normative behaviors of two or more different groups, thus putting bridges at an advantage knowing which behaviors are being adopted in different groups. This may enable them to be early adopters relative to one or another group. In addition, because bridging connects people to different norms, it may enable bridges to be more open to change and new ideas in general. Finally, bridging individuals may be less constrained than central members because they are not beholden to the norms of any one group (Burt, 1992; Valente & Fujimoto, 2010).

## Network-Level

Several network-level properties have been hypothesized to be associated with the diffusion of innovations or behavior change. Network-level indicators are measures that characterize the whole network with a single indicator. Indeed, many of the personal network weightings we discussed above are also network-level ones. The personal network influences based on alter characteristics such as reciprocity or Simmelian ties can be summarized for the entire network by taking the average (or some other statistic) of the rates for individuals. For example, the average level of reciprocity, tie strength, and number of Simmelian ties in the network would be network-level metrics. Here, we consider three network-level metrics: density, centralization, and clustering.

Note that when shifting from individual or personal network influences to network-level ones, we shift the unit of analyses from the individual to the whole network or system. This is important for several reasons: (1) network level properties can influence the rate and prevalence of diffusion within a single system, (2) to compare processes across systems such as when collecting the same data across schools or organizations, and (3) the network-level measures may condition personal network influences as we discuss later.

### Density

Density has been hypothesized to accelerate diffusion because there are more links for information and influence to spread practices (Valente, 1995). In one empirical study, however, density was negatively associated with reports to adopt evidence-based programs (Valente et al., 2007). Some minimum density no doubt is necessary for diffusion to occur, and simulations have shown that densities between 15% and 25% can be structurally unstable (Valente, 2010). So, both theory and data seem to suggest that density may accelerate or retard diffusion.

### Centralization

Centralized networks are thought to be more efficient because the central nodes can coordinate and control the network more effectively than in a decentralized network. Conversely, centralized networks may be less sustainable because the majority of network members do not occupy central positions and hence may feel left out of decisions and less engaged in network activities. It may be that centralized networks are good for diffusing easy-to-adopt behaviors and those that are not controversial, whereas decentralized ones are better when the innovation is hard-to-adopt or controversial.

### Clustering

Clustering is the rate at which network ties are contained within groups rather than between them. These groups may be defined by individual attributes (e.g., grade in school in a school network) or may be derived from the network data based on various algorithms that can find such clusters. Clustering is sometimes measured with a statistic called modularity, which calculates the ratio of links within groups to links between groups. High rates of modularity (Girvan & Newman, 2002) might be thought to slow diffusion because it indicates high rates of clustering in the network, which would facilitate diffusion within clusters, but inhibit it between clusters.

In addition to these network metrics, there are other considerations that can affect the rate of diffusion. One is the community’s perception of the innovation: Is the new behavior compatible with past practices or does it represent a threat to the status quo and consequently is viewed with concern or suspicion? Becker (1970) classified innovations as low vs. high adoption potential. Also, considerable research has documented the role of perceived characteristics of the innovation such as its relative advantage, compatibility, trialability, complexity, observability, radicalness, and cost.

### Personal, Positional, and Network-Level Interactions

Similar to the way cultural and artefactual characteristics affect the way individual and positional influences operate, network-level metrics such as density, centralization, and clustering create macro-level environments within which the micro-level effects discussed above take place. In the section on centrality above, it was noted that leaders may be early adopters of behaviors that are (1) considered normative or (2) ones which central members may reasonably believe will become widespread. Becker (1970), in his influential early study of network influences on diffusion, posited that central members adopt early when the behavior is compatible with the community, but delay adoption if the innovation is incompatible or for one that may have high uncertainty. This centrality–compatibility interaction is perhaps the most obvious and best-tested interaction between network effects at the micro- and macro levels, but there are potentially many others.

### Prevalence

Exposure, perceived exposure, and threshold effects may all vary depending on the current prevalence of the behavior. For example, community members may have high thresholds early in the diffusion process because they are uncertain as to whether the practice will become widespread and/or normative. Thus, it may take high exposure to the innovation before a person is willing to adopt it, yet there are only a few adopters, so exposures are low on average. As diffusion occurs, exposure to the innovation increases; coincidentally many people may lower their thresholds, requiring few prior adopters to adopt.

Tie strength is also likely to be affected by prevalence such that it may take strong ties to persuade a person to adopt a new behavior when prevalence is low (early in diffusion) but weaker ties may suffice when prevalence is high. Indeed, potentially all of the individual and positional influences may vary by prevalence because norms and what is considered normative will vary by prevalence; there is a potentially complex dynamic interplay between prevalence and network influences, however defined.

### Centralization

Centralization is also likely to affect individual and positional influences. For example, being central in a centralized network has different connotations than being central in a decentralized one. Central actors in centralized networks have considerable power and control over the network because they occupy the central position and the network is focused around them. Being the most central in a decentralized network does not provide much of a position of advantage relative to learning about new practices in the network or being able to control others’ actions. Being central in a centralized network is also likely to create increased social pressure on central actors because any action can risk losing centrality; conversely, a central actor in a decentralized network may be aware that they are not in such a privileged position and so strive to enhance their central status. Centralization may also affect other individual and positional measures but there is little evidence to indicate how.

### Clustering

Similar to the centrality–centralization interaction, occupying a bridging role may be particularly important when the network is highly clustered. Bridging individuals occupy positions in which they connect subgroups or cliques in the network. Therefore, bridges in a highly clustered network can act as gatekeepers between groups and in some settings may be critical to organizational functioning. For example, two organizations that merge to form a new company may have few bridges that connect members of the two groups and so those that do can be critical to communications within the organization.

Clearly this list of micro- and macro-level influences and interactions is not comprehensive, but instead is meant to stimulate studies comparing these mechanisms. In addition, these different structural mechanisms may be overlaid with specific interpersonal interactions such as persuasion, information sharing, modeling, and so on that indicate how influence occurs. Social psychological influence mechanisms such as cognitive processing, group identification, or classic persuasion have not been discussed, but surely the network structural mechanisms articulated here may interact with them. Table 2 summarizes the influence effects discussed in this paper.

## Contagion Redux

This article began by stating that understanding contagion is important and it is often based on direct contact between peers. Social network theory provides an expanded list of the ways interpersonal influence can be conceived and modeled. When we invoke contagion as a mechanism, we usually think of a disease or a behavior being contagious such that it spreads with a certain velocity or transmissibility. In other words, contagion gives agency to the disease, virus, illness, or behavior that is spreading. When we say an innovation is contagious, it signifies that the product has attributes that will induce individuals to readily accept and use it. In contrast, when we invoke interpersonal influence explanations, it conjures thoughts of the actors, the ability to persuade, or be persuaded by one another. Interpersonal influence gives agency to the actors, not the product.

Imagine an infectious disease such as the flu or COVID-19 spreading in a community (unfortunately it is not hard to do). To describe this process, we may say that this year’s flu is highly contagious. In some cases, contagion explanations match with the descriptions of behavior change well. Exposure is clearly analogous to contagion: being in contact with adopters can result in becoming an adopter, and the more exposures one has to other adopters, the greater the likelihood of change (becoming an adopter or infected). The concept of thresholds seems to apply as well: some people become infected with only one exposure, yet others seem entirely resistant to some diseases or behavior changes. But the parallels seem to stop there.

It is hard to imagine how Simmelian ties may affect contagion because the connection between two alters does not affect the transmissibility of the disease. Similarly, for perceived use, thinking an alter has a cold is unlikely to affect whether ego develops one (although presumably one could lower their immunities if they think they are bound to become sick because their roommate is sick). On the other hand, if someone has a cold, you may avoid interacting with them for fear of catching it, just as some people avoid smokers or drinkers.

Indirect influences do not have a contagion analog, nor does SE. For example, contagion would not be invoked to explain the influence of network indicator weights since the innovation has not changed simply because the influencer has high centrality for example. In other words, it seems that when the characteristics of the actors are what varies we move from a contagion explanation to an influence one.

When behavior change occurs as a result of an actor’s network position, there may be both contagion and social or interpersonal influence mechanisms occurring. For example, if central members are early adopters, they may do so because they have more contacts and this exposes them to the innovation earlier than less central members. Similarly, central members can be at greater risk for infection, contagion (Christakis & Fowler, 2010; Garcia-Herranz et al., 2014). On the other hand, if central members adopt early because they perceive the innovation to be culturally compatible and will likely become widespread; it is not contagion that is driving change but instead actor-defined and socially defined mechanisms.

At the network level, both contagion and influence mechanisms are invoked although in slightly different ways. Contagion is invoked when we make statements such as: tuberculosis spread rapidly in the tenement buildings because they were so crowded (density). Influence is invoked when we make statements such as: the new billing system spread rapidly in the organization because everyone knew one another (also density). So, it seems network-level factors can take on both contagion and interpersonal influence descriptions.

For the individual and positional network-level interaction influences, contagion effects also apply. For example, it has been shown that central individuals, due to their greater exposure, become infected earlier than less central individuals (Christakis & Fowler, 2010). Here, it is the positional influence acting through the interpersonal exposure influence. Further, there is a positional and network-level interaction that can drive contagion as central individuals in centralized networks are likely to be infected earlier than central individuals in decentralized networks. We now have positional influence being conditioned on the network-level measure affecting rates of direct exposure.

The interactions of individual, positional, and network-level interactions in the context of behavioral adoptions can be further complicated by individual characteristics. Factors such as perceptions of uncertainty, cultural norms, perceived characteristics such as relative advantage, complexity, trialability, cost and so on can affect decision-making. For behavioral influences, incorporating the network interactions can further shift agency away from the product/behavior and closer to individual actions and social ones. Thus, agency is at the intersection between individual motivations and how they are shaped by the network-level environment.

There are research implications from the observations made in this paper. First, there are lots of ways to assess potential network influences beyond mere exposure. Second, behavior change theory should guide the specification of these network influences. Third, there is much theoretical development to pursue, particularly in the realm of individual network-level interaction effects. It is hoped that by explaining the way network structural configurations affect diffusion, behavior change, and, in some cases, disease spread, others will be encouraged to employ and test a broader array of network effects. It is also hoped that researchers now appreciate the distinction between contagion and interpersonal influence when making statements of how change occurs.

## Figures and Tables

**Table 1.** Adjusted odds ratios for becoming a smoker over a two-and-a-half-year period using different network weighting mechanisms.

Influences	Smoking at time 2
Individual	
Exposure percent	3.69**
Exposure count	1.20
Perceived use	2.34**
Indirect ties-2 step	3.56**
SE	0.26*
Tie characteristics	
Simmelian ties	4.29**
Network density	2.94**
Alter characteristics	
Network indicator weights – Indegree centrality	2.95**
Alter attributes-same sex	3.42**
Joint participation-same team	1.39

SE, structural equivalence.

* p < 0.05;

** p < 0.01.

Code and data can be found here: https://github.com/gvegayon/sns-exposures/blob/master/data/exposures.R

NB: Regression includes being female, number of rooms in household, academic grades, parental smoking, sibling smoking, being Hispanic/Latino, and fixed effects for school. Analysis conducted using the NetdiffuseR package in R.

**Table 2.** Summary of example social network influence mechanisms.

Personal network		Positional	Network level	Interactions
1) Exposure	6) Tie strength	1) Central	1) Density	1) Exposure and density
2) Perceived use	7) Reciprocity	2) Peripheral	2) Central-ization	2) Central actors in centralized networks
3) Thresholds	8) Simmelian ties	3) Bridge	3) Clustering	3) Bridging actors in clustered networks
4) Indirect ties	9) Density			
5) SE	10) Alter attributes			
	11) Joint participation			
	12) Network indicator			

SE, structural equivalence.
